# Current and Future Concepts for the Treatment of Impaired Fracture Healing

**DOI:** 10.3390/ijms20225805

**Published:** 2019-11-19

**Authors:** Carsten W. Schlickewei, Holger Kleinertz, Darius M. Thiesen, Konrad Mader, Matthias Priemel, Karl-Heinz Frosch, Johannes Keller

**Affiliations:** Clinic of Trauma, Hand and Reconstructive Surgery, University Medical Center Hamburg-Eppendorf, 20246 Hamburg, Germany; c.schlickewei@uke.de (C.W.S.); h.kleinertz@uke.de (H.K.); d.thiesen@uke.de (D.M.T.); k.mader@uke.de (K.M.); priemel@uke.de (M.P.); k.frosch@uke.de (K.-H.F.)

**Keywords:** fracture healing, non-union, osteoanabolic molecules, biomaterials

## Abstract

Bone regeneration represents a complex process, of which basic biologic principles have been evolutionarily conserved over a broad range of different species. Bone represents one of few tissues that can heal without forming a fibrous scar and, as such, resembles a unique form of tissue regeneration. Despite a tremendous improvement in surgical techniques in the past decades, impaired bone regeneration including non-unions still affect a significant number of patients with fractures. As impaired bone regeneration is associated with high socio-economic implications, it is an essential clinical need to gain a full understanding of the pathophysiology and identify novel treatment approaches. This review focuses on the clinical implications of impaired bone regeneration, including currently available treatment options. Moreover, recent advances in the understanding of fracture healing are discussed, which have resulted in the identification and development of novel therapeutic approaches for affected patients.

## 1. Clinical Challenge of Impaired Fracture Healing

In the majority of patients with a long bone fracture, the fracture ends meet to reform a mature and mechanically stable bone: Especially in the case of long bone fractures, this is usually achieved by surgical state-of-the-art treatment. In a significant number of cases, however, fractures do not heal and show no or insufficient signs of union [1]. The development of non-union is a devastating complication and a challenge for both patients and physicians. The treatment of non-unions often requires several revision surgeries, which result in prolonged treatment that affects the patient’s mental and physical health and thus represents a major socio-economic burden [2,3]. Delayed unions show a clinically and radiologically prolonged bone healing process which usually resolves once adequate measures are taken, whereas definite fracture non-union occurs when a fractured bone fails to heal at all. To date, several definitions for non-union exist. For example, the European Society of Tissue Regeneration in Orthopedics and Traumatology (ESTROT) defines a non-union as a fracture, which will not heal or consolidate without further intervention, regardless of the previous treatment duration [4]. According to the U.S. Food and Drug Administration (FDA), a non-union is defined as a fracture, which is at least nine months old and has not shown any signs of healing for three consecutive months [5]. Clinically, non-unions are classified according to their morphology and radiographic appearance in hypertrophic and atrophic non-unions [6]. Their appearance correlates with characteristic pathophysiologic processes at the fracture site. Hypertrophic non-unions are characterized by vital, regenerating bone tissue with good blood perfusion of the fracture ends and the availability of necessary molecular mediators, progenitor cells, osteoconductive matrix, and immunoregulatory cells among others. However, hypertrophic non-unions fail to heal because of insufficient mechanical stabilization and consecutive excessive mobilization, which results in an increased callus formation trying to compensate for the lack of stability. Despite high regenerative potential, fracture union is thus prevented by the instability and high mobility of the fragments. In contrast, atrophic non-unions are characterized by the lack of regenerative potential, which is often associated with insufficient blood supply of the fracture ends and the surrounding soft tissue, or low-grade infections of the fracture site. Despite adequate mechanical fixation, bone regeneration fails in these cases and the fracture ends atrophy.

The reasons for the formation of non-unions are complex and manifold (Table 1). The prevalence regardless of individual risk factors is about 10–15% [7]. Above all, higher age, smoking, vascular disease, former fractures, diabetes mellitus and metabolic diseases are among the primary risk factors of impaired bone healing. In addition to the severity and complexity of the fracture, pronounced soft tissue trauma and inadequate blood supply is also predisposing. Non-unions due to bacterial infection of the fracture site often develop after high-energy trauma with open fractures and severe soft tissue damages. In this case, low-grade infections are of increasing clinical relevance, as they are suspected to cause non-unions in a large percentage of affected patients and often are difficult to detect clinically, even with state-of-the art diagnostics methods.

## 2. Basic Biological Principles of Bone Regeneration

Even though stiff and sturdy, bone tissue is biologically very active and constantly remodeled through the balanced activities of bone-resorbing osteoclasts and bone-forming osteoblasts. Similar to bone remodeling in healthy skeletal tissue, bone regeneration represents a complex process which basic biologic principles have been evolutionary conserved over a broad range of different species. It is known that bone represents one of the few tissues that can heal without forming a fibrous scar. As such, the process of fracture healing is considered to recapitulate bone development and resembles a unique form of tissue regeneration. In this context, callus remodeling represents an important aspect of fracture repair, resulting in bone quality and strength comparable to the pre-fracture state [9].

Fracture healing is traditionally subdivided into various biological phases which follow a chronological order. These particular phases help to understand the biology of fracture repair on a mechanistic level, however they cannot be strictly separated as they occur simultaneously at different sites and at different rates throughout the fracture callus. In general, tissue healing is characterized by an inflammatory, a proliferation/differentiation, as well as a remodeling phase. The same applies for fracture healing but has to be extended by matrix ossification, which occurs between the proliferation/differentiation and the remodeling phase [10].

### 2.1. Hematoma Formation and Inflammatory Response

As a first consequence of fracture and vascular disruption, bone marrow and vascular leakage create a local hematoma containing bone and immune cells [11]. The formation of the fracture hematoma is essential for adequate bone healing, as it is characterized by high osteogenic potency [12,13]. This characteristic is mainly attributed to cells first described in the 1962 as unspecialized osteoprogenitor cells, nowadays known as mesenchymal stem cells (MSC) [14,15]. They possess osteogenic, chondrogenic and adipogenic differentiation potential [16]. Additional cells found abundantly in the fracture hematoma include platelets and macrophages. Of note, particular cytokines which activate the clotting cascade in the fracture hematoma also activate local phagocytic effector cells such as macrophages which remove bone and tissue debris at the fracture site [17]. Although temporary, local hypoxia causes bone and soft tissue necrosis at the fracture site, cytokine release and migration of pro-inflammatory immune cells. This creates an inflammatory environment that is characterized by increased local blood flow and vascular permeability, promoting further influx of pro-inflammatory cells and increased cytokine production [10,11].

Pro-inflammatory cytokines in the innate response to a fracture include interleukin (IL) -1β, IL-6 and tumor necrosis factor-α (TNF-α) [18]. While IL-1β and TNF-α peak early at 4 h after fracture, IL-6 peaks at 12 h and a second TNF-α peak is seen at 24 h [19]. Deficiency in IL-6 was found to be associated with delayed callus mineralization and increased cartilage and collagen content accompanied by a decreased osteoclast activity, suggesting impaired remodeling [20]. Additionally, reduced callus strength and impaired osteoclastogenesis two weeks after fracture were reported in IL-6 deficient mice [21]. Both studies found the effects of IL-6 deficiency to be of transient nature [20,21]. TNF-α is essential for adequate enchondral ossification by mediating chondrogenic differentiation and enchondral tissue resorption. Impaired TNF-α activity is associated with reduced chondrocyte apoptosis and pro-resorptive cytokine production [22]. Further, TNF-α was also shown to promote muscle-derived stomal cell (MDSC) migration and osteogenic differentiation, summing up to an accelerated fracture healing. However, impaired fracture healing was seen in the presence of high concentrations [23].

### 2.2. Proliferation and Differentiation

While the above described inflammatory processes subsequently decline, the reparative phase begins in parallel by the formation of granulation tissue. As mesenchymal stem cells emerge and differentiate into fibroblasts, they produce extracellular matrix proteins such as collagens type I and II [7,24]. Due to local hypoxia, the formation and invasion of blood vessels is promoted. MSC differentiate into chondrogenic and osteogenic cells and form soft callus tissue, which provides a first degree of mechanical stability.

On a molecular level, these reparative processes are mediated by particular growth factors like transforming growth factor-β (TGF-β), bone morphogenic proteins (BMP), fibroblast growth factor (FGF), insulin like growth factor (IGF) and platelet derived growth factor (PDGF). TGF-β acts as pleiotropic growth factor stimulating undifferentiated MSC proliferation while BMPs promote MSC differentiation to chondrocytes and osteoblasts. FGF and PDGF act mitogenic on MSCs and osteoblasts. While FGF is critical for angiogenesis, PDGF also acts in macrophage chemotaxis. IGF promotes proliferation and differentiation of osteoprogenitor cells [25].

### 2.3. Ossification

Since the soft callus itself only provides basic mechanical stability, it is subject to ossification that is achieved through incorporation of calcium phosphate into the extracellular matrix. Ossification is distinguished in intramembranous and enchondral ossification which occur under different conditions and are likely to occur simultaneously at different zones in the fracture site [7]. In intramembranous ossification, bone develops without cartilaginous intermediates. Upon stimulation, MSC differentiate into osteoblasts that produce the extracellular matrix primarily composed of type I collagen and other osteoblast-specific proteins including osteocalcin. The extracellular bone matrix then calcifies through the activity of osteoblasts which deposit calcium phosphate crystals within the extracellular matrix. As osteoblasts become “trapped” in the calcified extracellular matrix, they terminally differentiate into mechanosensitive osteocytes. In contrast to intramembranous ossification, bone healing is achieved through cartilaginous intermediates in enchondral ossification. Here, chondrocytes differentiated from MSC produce a chondrogenic matrix that bridges the fracture. Eventually, chondrocytes undergo apoptosis, while the cartilaginous tissue rich in collagen type II is enzymatically degraded and replaced by osseous tissue containing predominantly collagen type I [26].

### 2.4. Remodeling Phase

Independent of whether bone is restored via intramembranous or enchondral ossification, mechanically inferior woven bone is created at first. Woven bone is characterized by a random organization of collagen fibers and mineralized tissue. In order to generate mechanically stable lamellar bone, the woven bone is subsequently remodeled to form mechanically competent lamellar bone. Similar to healthy bone of which 10% is replaced and remodeled each year in humans [27], the transition of woven to lamellar bone is mediated by the balanced activity of osteoblasts, osteocytes and osteoclasts. This process is associated with final changes in bone architecture and allows the former fracture site to adapt to the current mechanical demands.

### 2.5. Mechanical Aspects of Fracture Healing

The outcome of bone regeneration depends on both the biological and the mechanical environment. The mechanical environment primarily influences whether bone heals primary (direct) or secondary (indirect). Primary bone healing can be observed in anatomically reduced fractures with rigid fixation and interfragmentary compression. In this case, the fracture heals only through bone remodeling, without the formation of a fracture callus [9,28]. In contrast, secondary bone healing describes bone tissue formation via intramembranous and enchondral ossification of callus tissue, and represents the most common form of bone regeneration in clinical practice [29]. Secondary fracture healing applies to fractures treated without surgery as well as fractures treated with intramedullary nails or external fixation that do not achieve the rigidity and interfragmentary compression required for primary bone healing [30].

As both, intramembranous and enchondral ossification, are observed in secondary bone healing, the degree of fixation determines the principle way of ossification. Experimentally, Thompson et al. could show that externally stabilized fractures healed primarily via intramembranous ossification in a standardized mouse model of tibial fracture, while the expression of collagen type IIa pointed towards enchondral ossification in non-stabilized fractures. Moreover, the authors concluded that the mechanical environment influences cell fate and thus the course of ossification as soon as day 4 in their mouse model, highlighting the importance of the early fracture hematoma for successful bone regeneration [31].

## 3. Current State-of-the-Art Therapy for Impaired Fracture Healing

### 3.1. Case History, Physical and Radiological Examination

Regardless of past, current or future treatment concepts, a detailed medical history of the patient and the individual case is paramount for an optimal and individual therapy of non-unions. For this purpose, it is important to establish a chronological list of the previous course of treatment with all therapeutic interventions, as well as risk factors, pre-existing illnesses and long-term medication. Often neglected is the thorough study of medical and surgical reports including microbiological and histological findings, the previous drug history and the chronological course of the infection parameters.

Clinical symptoms of non-union include persistent, often relapsing stress-related pain in the area of the former fracture. Malposition or load-dependent and reproducible instability may be present. Clinical examination should include a detailed inspection of the soft tissue, the scars and a blood flow assessment at the affected limb. In case of evidence for impaired vascularization, CT-angiography is indicated. Moreover, a detailed study of existing radiographic imaging should be performed as it is crucial not only to review the latest radiographs, but also the initial accident images, the intraoperative fluoroscopy and the follow-up images during the healing process chronologically. Only in combination of all relevant findings it is possible to classify a non-union as precisely as possible and to establish an individual treatment strategy. With correlating clinical symptoms, a whole-limb radiograph should be performed in order to detect malposition or axial deviation. For accurate analysis and pre-operative planning, a computed tomography is of utmost importance. In selected cases, further imaging using contrast-enhanced CT, magnetic resonance imaging, angiography or PET-CT provide further information regarding osteomyelitis, blood perfusion, and soft tissue coverage.

### 3.2. Current Treatment Concepts

*Restitutio ad integrum* is the intended goal of any non-union treatment. In addition to achieving a pain-free and load-stable situation, the chosen approach ideally results in full restoration of the axis and length of the affected limb, as well as in complete healing of the bone defect. Apart from various surgical approaches, conservative treatment options are also available in individual cases, e.g., in delayed unions with prior sufficient fracture immobilization through stable osteosynthesis. In the case of non-unions with no signs of osseous consolidation, however, revision surgery is usually indicated. The treatment is usually based on the “Diamond Concept” [32], a conceptual framework which gives equal importance to mechanical stability, the biological environment, adequate bone vascularity and the physiological state of the patient. Depending on the pathology, individual or combined measures are possible, and two-stage procedures may be necessary, for example in the case of infected or atrophic non-unions, sequesters, and critical-size bone defects. As a general guideline, Calori et al. presented a novel score for the classification and treatment algorithm of non-unions [32]. Based on a calculated score, therapeutic recommendations for the treatment of non-unions are derived, which take into account the localization of the injury, soft tissue damage, bone quality and the individual risk of the patient.

### 3.3. Conservative Therapy

Conservative treatment of delayed and non-unions requires sufficient mechanical stability as well as an intact biological environment at and around the fracture site. Therefore, conservative therapeutic approaches are mainly considered in early phases of non-union treatment. In addition to stimulate fracture healing through an increased mechanical strain (e.g., dynamization of intramedullary fixation, increased weight bearing), low-intensity pulsed ultrasound (LIPUS) or extracorporal shock wave therapy (ESWT) may be applied locally [33,34]. Depending on the treatment units, the time frame of the treatment with LIPUS is 3–6 months with a daily application of 20 min [35]. Criteria for a possible treatment of non-union with LIPUS and ESWT include sufficient mechanical stability of the fracture fixation, no evidence for high- or low-grade infection, and a defect size of less than 10 mm. Nevertheless, a systematic review and meta-analysis of non-unions treated with LIPUS showed a healing rate of hypertrophic non-unions older than 8 month of up to 84% [36]. An average treatment success with LIPUS of >80% shows a comparable success rate with the surgical treatment of non-infected non-unions, while the authors state that LIPUS could be most useful for patients with increased surgical risk [36].

### 3.4. Surgical Therapy

While the efficacy of LIPUS and ESWT require further clinical research, to date the standard treatment of fracture non-union is surgical. The surgical intervention begins with the careful exposure of the fracture site and the debridement of sclerotic edges in order to obtain a vital and bleeding surface. Thereafter, the intramedullary cavities of fragments may be opened in order to facilitate the blood flow. As vital fracture ends are aligned rigid fixation may be performed. In many cases, resection of the malunion consisting of fibrous, often atrophic tissue, results in bone defects, which require bone grafting to bridge the fracture ends and to facilitate bone healing.

Despite all efforts in the research of artificial bone substitutes, the harvest and transplantation of autogenous bone from the iliac crest still represents the “gold standard” to support bone healing in non-unions as it combines osteogenic, osteoinductive and osteoconductive properties. However, autografts are only limited available and accompanied with high morbidity during harvest, including wound infection and postoperative pain [14,37]. An innovative intervention to obtain autologous spongiosa is the Reamer-Irrigator-Aspirator System (RIA). The RIA technic enables the collection of almost 80 cm^3^ of bone marrow aspirate and has gained wide acceptance in the treatment of non-unions over the last years [38,39]. Studies showed significantly higher concentrations of growth factors relevant for bone healing with significantly lower complications for autologous bone graft harvesting via RIA compared to iliac crest grafts [40,41]. Even though having similar osteogenic and osteoinductive properties as iliac crest bone grafts, the RIA bone marrow aspirate lacks osteoconductive properties as it lacks intrinsic biomechanical stability [42].

Allogenic bone grafts provide a relatively safe alternative as autografts have limited availability and harvesting is associated with longer operation time and donor site morbidity. Allografts are usually used as cancellous bone chips providing some degree of structural strength. Due to their porous nature, ready-to-use bone allografts have very good osteoconductive properties. However, processing in terms of sterilization and storage causes loss of osteogenic and osteoinductive capability [42]. While the use of allografts was shown beneficial in the treatment of acute metaphyseal fractures, the results for long bone fracture non-unions are disappointing [43,44,45]. Finally, demineralized bone matrix (DBM) can be obtained from allograft bone by acid extraction [46]. This process allows the conservation of type I collagen and non-collagenous proteins such as BMPs, TGFs, IGFs and FGFs exerting osteoinduction. Even though mechanical support is limited, DMB provides an osteoconductive scaffold. Hierholzer et al. could show DBM to achieve results comparable to those of cancellous autografts in humeral shaft fracture non-union treatment [47]. Osteoinductivity differs among manufacturers and extraction regimes suggesting a major influence of processing on the osteoinductive potential [48,49].

**Figure 1:**
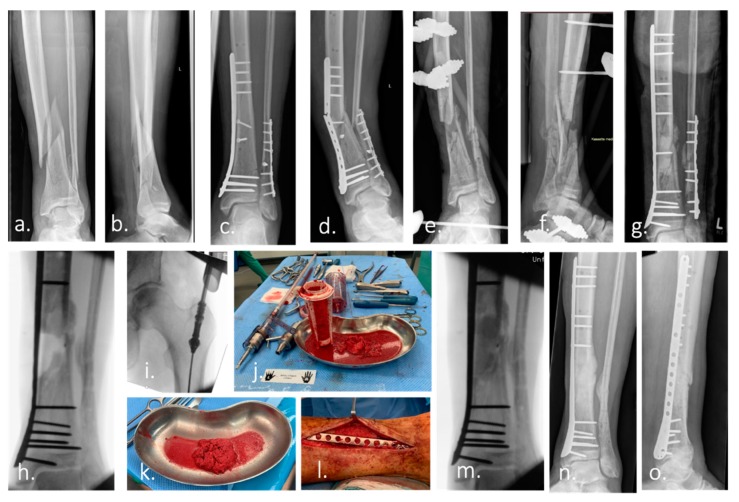
Case example of infected non-union treated with Masquelet technique and Reamer–irrigator–aspirator. (**a**,**b**) Ap and lateral radiograph of a multi-fragmentary Gustilo–Anderson Type II open tibia and fibula fracture of a 74-year-old man. (**c**) Ap radiograph after surgical treatment. (**d**) 7 month after surgery: re-presentation of the patient with an infected non-union of the tibia with re-fracture and failure of the osteosynthesis material. (**e**,**f**) Postoperative Radiograph of the lower leg (ap and lateral): Condition after removal of the broken osteosynthesis material. Large, bone defect of the tibia after extensive debridement, reaming of the medullary canal, radical resection of the non-union and temporary immobilization with an external fixator. The microbiological samples taken intraoperatively revealed the presence of Staphylococcus caprae. Antibiotic therapy was carried out according to the recommendations of the interdisciplinary infection board. (**g**) Re-osteosynthesis of tibia and fibula after resolution of infection. Temporary treatment of the bone defect with a cement spacer to induce a vascularized foreign-body membrane (Masquelet membrane). (**h**) Intraoperative radiograph after removal of the cement spacer 2 months after implantation. (**i**–**k**) Harvest of autologous bone graft (bone marrow, morselized bone) from the femur using the Reamer–irrigator–aspirator (RIA). (**l**,**m**) Picture and intraoperative radiograph after filling of the bone defect with autologous bone. (**n**,**o**) Ap and lateral radiograph of the left lower leg demonstrate healing of the bone defect 4 month after surgery.

**Figure 2:**
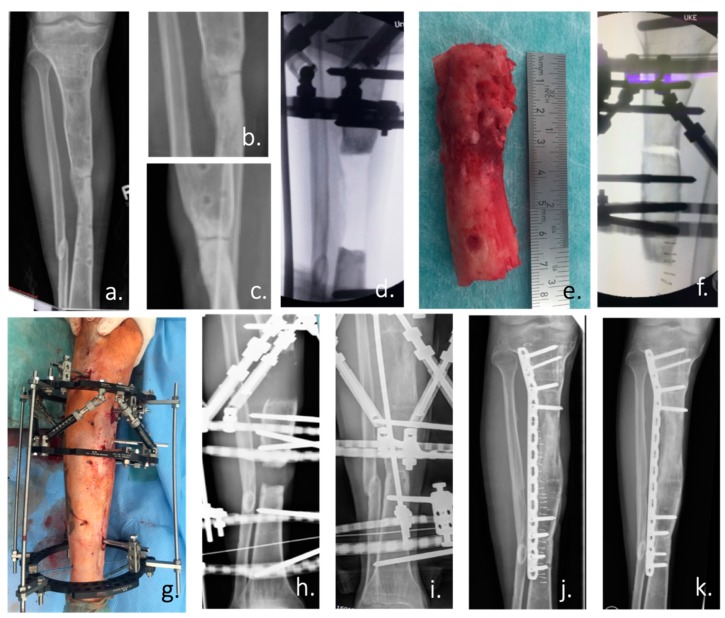
(**a**) Ap radiograph of the right lower leg of a 24-year-old man with an atrophic non-union of the tibia after an open fracture in Albania 5 years ago. (**b**,**c**) Enlargement of the non-union in an ap and lateral radiograph. According to the patient’s history: condition after 4 surgical trials in 3 countries. (**d**) Intraoperative ap radiograph after positioning of the Taylor Spatial Frame (TSF) external fixator and radical resection of the atrophic non-union. (**e**) Resected tibia non-union with atrophic bone over a length of 7 cm. (**f**) Intraoperative positioning of the tibia and the TSF for the planned bone segment transport by callus distraction. (**g**) Postoperative picture of the attached TSF. (**h**) Radiograph of the tibia during the distraction process (0.5 mm/day over a period of 140 days) with constant substitution of calcium and vitamin D. (**i**) X-ray of the tibia after the end of the distraction with already clearly visible bone formation. (**j**) Postoperative radiograph after removal of the ring fixator and angular stable plate osteosynthesis. (**k**) Radiological control at the end of treatment with healed bone defect 3 months after TSF removal.

Based on the different underlying causes described above, the different forms of non-unions require specific surgical strategies. In case of hypertrophic non-unions, a more rigid fixation is required to allow proper fracture union through replacement of the osteosynthesis and/or additional stabilization. Apart from locking plates, intramedullary nail osteosynthesis is often used, especially in the shaft area, which provides the advantage of early patient mobilization under full load weight bearing. In contrast, atrophic and defect non-unions are often associated with an impaired biological environment at the fracture site due to metabolic, vascular or infectious co-morbidities of the patient. Complete and rigorous resection of the non-union is required, as incomplete removal of atrophic tissue to avoid larger bone defects usually leads to failure of fracture union and is associated with poor outcome. Following resection, reconstruction of the defect zone may be performed through bridging with autologous spongiosa or, in the case of larger defects, additional application of an osteoconductive scaffolds (e.g., of allogenic bone graft). If the bone defect is critical (>2 cm or >5 cm^3^), or if infection is suspected, a multi-stage procedure with temporary implantation of a cement spacer to induce a Masquelet membrane is recommended (Figure 1). The cement spacer is removed after an interval of 6–8 weeks, and autologous spongiosa is filled into the resulting lumen of the Masquelet membrane, allowing ossification and subsequent bridging of the bone defect [50,51]. In the case of very large defects, Masquelet bone reconstruction, bone segment transport by callus distraction with a ring fixator (Figure 2), or vessel-guided bone grafting must be performed.

Similar to atrophic or defect non-unions, infected non-unions usually require a multi-step and often an interdisciplinary approach. The initial surgical treatment is used exclusively to eliminate the infection and requires the complete resection of the infected and avital bone. Surgical debridement must be executed rigorously and address all infected tissue to avoid repeated surgery due to persistent infection. As part of the primary procedure, histological and microbiological samples should be obtained for antibiogram/resistogram adjusted antibiotic therapy. Only after complete resolution of infection, the reconstruction of the bone defect for successful healing of the non-union can be performed in a secondary surgery. Here, reconstruction of the bone defect is carried out according to the standards for atrophic non-unions as described above.

## 4. Novel Approaches for Impaired Fracture Healing

Although non-unions can be treated successfully in the majority of cases using the approaches described above, the often highly specialized and individual treatment concepts are expensive, time-consuming and a large burden to affected patients. Therefore, it is essential to establish novel therapeutic approaches, which either prevent impaired bone healing or facilitate bone regeneration when non-union has occurred. These include, but are not limited to, molecular mediators which promote the formation of new bone, as well as bone grafts or substitute materials which serve as a scaffold for osseous bridging of bone defects.

### 4.1. Molecular Targets for Local and/or Systemic Application

**Figure 3:**
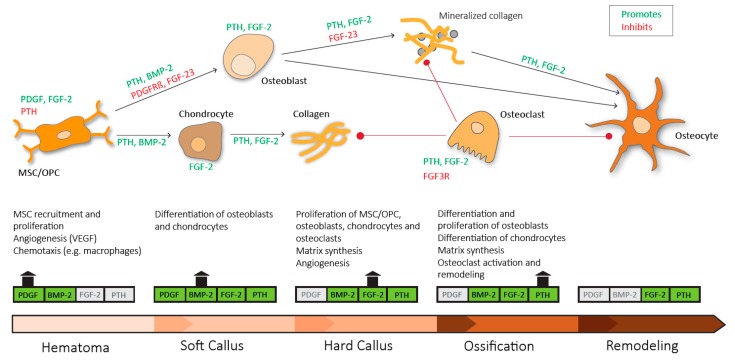
Sequential need for growth hormones and their effect on fracture repair. Platelet derived growth factor (PDGF) acts in recruitment and proliferation of mesenchymal stem cells/osteoprogenitor cells (MSC/OPC), exerts positive effects on angiogenesis via vascular endothelial growth factor (VEGF) and acts chemotactic on immune cells. Platelet derived growth factor receptor β (PDGFRβ) signaling suppresses osteogenic differentiation. Bone morphogenic protein-2 (BMP-2) promotes osteoblastic and chondrocyte differentiation. Fibroblast growth factor-2 (FGF-2) acts mitogenic on MSC/OPC, osteoblasts, chondrocytes and osteoclasts, and enhances matrix synthesis and angiogenesis. Fibroblast growth factor 23 (FGF-23) inhibits osteoblast differentiation and matrix synthesis. Fibroblast growth factor receptor 3 (FGFR3) signaling inhibits osteoclastic bone resorption. Parathyroid hormone (PTH) enhances differentiation and proliferation of osteoblasts, enhances differentiation of chondrocytes, enhances matrix synthesis and activates osteoclasts and bone remodeling.

Investigations in both humans and animal models have provided valuable insights into the pivotal pathways that regulate bone regeneration in health and disease. Although the role of many mediators expressed in cells of the fracture callus is not fully understood, research in the past decades has identified several key molecules, which may potentially be used to promote bone regeneration (Figure 3).

#### 4.1.1. Parathyroid Hormone

Parathyroid hormone (PTH) is a polypeptide hormone composed of 84 amino acids and is produced by the parathyroid glands. PTH regulates calcium hemostasis by increasing calcium resorption from bone and kidney directly as well as indirectly via vitamin D from the intestines [52]. In 1997, Dobnig et al. demonstrated that PTH could exert anabolic effects on bone when used in a pulsatile manner, while catabolic effects were observed during continuous exposure. Instead of using the 84 amino acid PTH, he used the amino terminal fragment known as teriparatide (PTH 1–34) [53]. Clinical trials proved both, PTH and teriparatide, beneficial in the treatment of postmenopausal osteoporosis by increasing bone mineral density (BMD), improvement of bone microarchitecture and a reduction in vertebral fractures [54,55]. A new agent, abaloparatide, a parathyroid hormone related protein (PTHrP) analogue, was shown to be beneficial in the treatment of postmenopausal osteoporosis and even superior to teriparatide in terms of vertebral fracture prevention, increase in BMD and overall response rate [56,57].

Intermittent PTH enhances bone formation by inhibiting proliferation of osteoblast progenitors determining them for osteoblast differentiation and inhibiting osteoblast apoptosis [58]. The positive effects of PTH/teriparatide on BMD and bone microarchitecture make its use in the treatment of impaired fracture healing appealing. A study with proximal tibial osteotomy in ovariectomized and sham rats could demonstrate an improved cancellous union rate with intermittent administration of teriparatide in either group [59]. Conflicting data was provided by Aspenberg et al. who could show a shortened time to cortical bridging in conservatively treated distal radius fractures in postmenopausal women with the injection of once daily teriparatide for 8 weeks at 20 µg, but not 40 µg [60]. A further study on unilateral pelvic fractures in osteoporotic women could show enhanced radiographic healing and improved functional outcome with once daily injection of 100 µg PTH. All pelvic fractures showed radiographic healing after 8 weeks in the PTH group while only 10% had healed in the control group at that time [61]. Despite these encouraging findings, dosing remains conflicting and requires further studies [60,61].

Bukata et al. addressed the use of teriparatide in cases of impaired fracture healing. 88% of the patients in their study either failed primary surgery, presented with delayed or non-union or were high-risk patients for impaired healing. Of 145 patients with complicated fracture healing, a 93% success rate with 12 weeks of adjuvant teriparatide treatment at 20 µg daily was observed. The authors propose teriparatide as a potential agent in complicated fractures and point out the need for prospective clinical trials [62]. Further clinical indications for the use of teriparatide might be found in arthroplasty. For example, teriparatide could reduce pain and increase periprosthetic BMD in the case of a 74-year old patient who had already undergone revision surgery and further surgery was no option due to comorbidities [63].

As of now, most studies on the use of teriparatide in fractures focused on the cancellous metaphyseal region. In human osteoporosis, Jiang et al. could show a positive effect of teriparatide on cancellous bone architecture as well as cortical thickness after 12-month of treatment [64]. Ogura et al. propose the PTH effect to be facilitated by different mechanisms in cancellous and cortical bone. The authors studied osterix expression as a marker for osteoprogenitor cell development, and osteocyte sclerostin expression. After 5 days of PTH treatment in rats, they observed an increased number of osterix-positive cells without a change in osteocyte sclerostin expression in cancellous bone. In cortical bone however, the number of sclerostin positive osteocytes was reduced [65]. Sclerostin is exclusively expressed by osteocytes, inhibits bone formation and is known to be suppressed by PTH [66,67]. However, cortical and cancellous bone formation is enhanced by PTH in a Wnt/β-catenin dependent manner after 28 days of application [68].

#### 4.1.2. Wnt Signaling and Sclerostin

**Figure 4:**
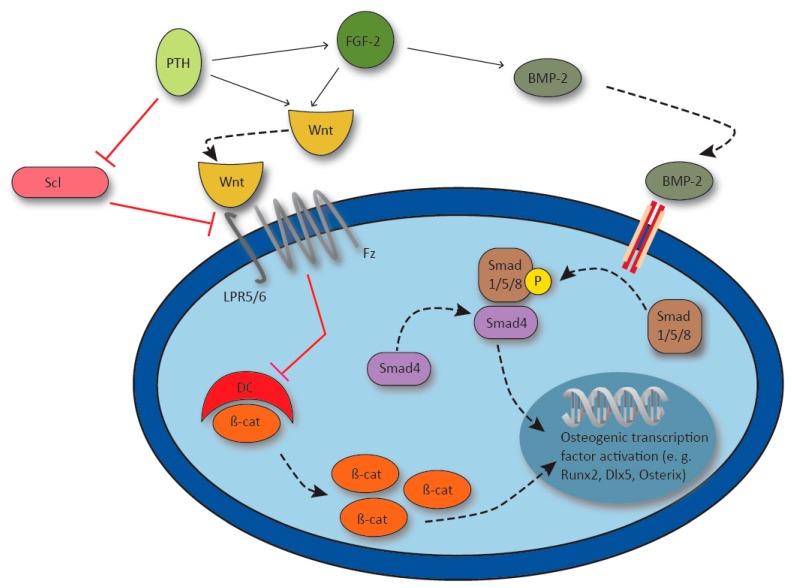
Synergy of parathyroid hormone (PTH), fibroblast growth factor 2 (FGF-2) and bone morphogenic protein-2 (BMP-2) in the activation of osteogenic gene transcription. PTH upregulates FGF-2, and PTH and FGF-2 synergistically activate Wnt signaling via lipoprotein receptor 5/6 (LPR5/6) and Frizzled receptor (Fz). Receptor activation inhibits the β-catenin destroy complex (DC) and β-catenin (β-cat) induces osteogenic gene transcription. FGF-2 enhances BMP-2 which activates transcription factor runt-related transcription factor 2 (Runx2), distal-less homebox 5 (Dlx5) and Osterix via Smad1/5/8 / Smad4 signaling. Osteocyte derived Sclerostin (Scl) inhibits Wnt signaling by LPR5 blockage and osteocyte Scl production is inhibited by PTH.

Wnt signaling is crucial in regulating diverse processes in tissue development and also plays a pivotal role in bone formation. Mechanistically, Wnt ligands bind to the Frizzled receptor (Fz) which forms a complex with the LDL receptor-related protein 5/6 (LRP5/6) co-receptor. Subsequently, LRP5/6 and Fz deactivate the β-catenin destruction complex, which leads to accumulation of β-catenin within the cell. β-catenin translocates to the nucleus, where it regulates the transcription of specific Wnt target genes and thus osteogenesis (Figure 4). For example, Wnt10b induces a shift towards osteoblastic differentiation by inducing the transcription factors runt-related transcription factor 2 (Runx2), distal-less homebox 5 (Dlx5) and osterix, while adipogenic transcription factors are suppressed [69]. Moreover, Cawthorn et al. demonstrated that the induction of osteoblastogenesis through Wnt6, Wnt10a and Wnt10b is dependent on β-catenin signaling [70]. In line with this, β-catenin levels in callus tissue were increased throughout the process of bone healing in mice as well as humans, and exerted different effects during the course of fracture healing. Wnt signaling was found to control the osteoblast/chondrocyte ratio in early stages, while it enhanced osteoblast differentiation and function as healing progressed. The authors point out that increased β-catenin levels at the early stage have a negative effect on healing time and emphasize the importance of adequate dosing [71]. Importantly, in a mouse fracture model, increased expression of Wnt4, Wnt5a/b and Wnt10b as well as nuclear β-catenin was achieved through the administration of teriparatide. Those findings were associated with enhanced callus formation and earlier chondrocyte hypertrophy, and indicate an involvement of Wnt signaling in the osteoanabolic effects of PTH [72].

As mentioned above, Wnt ligands induce various transcription factors. Runx2 is considered the master transcription factor in osteoblast differentiation, as it controls the osteoblast-specific cis acting element present in the promoter of several osteoblast specific genes and thus regulates the expression of major bone matrix proteins [73]. Genetically engineered bone marrow stomal cells overexpressing Runx2 accelerated healing in critical size femoral bone defects in rats compared to unmodified cells. However, after 12 weeks no difference in defect bridging or biomechanical function could be observed, pointing to a temporary benefit [74].

Sclerostin, a product of the SOST gene, is expressed in osteocytes and a well-established inhibitor of Wnt signaling and thus, bone formation [67] (Figure 4). Sclerostin has been described as a direct inhibitor of LPR5, since sclerostin inactivation leads to the same bone phenotype as activating mutations of LRP5 with enhanced bone growth known as sclerosteosis [75,76]. In osteoporosis, one of the most prevalent bone diseases worldwide, a monoclonal antibody inhibiting sclerostin known as romosozumab, was shown to reduce the risk for vertebral fractures in postmenopausal women within a 12 months period [77]. As a potential therapeutic agent in osteoporosis and thus, also impaired bone healing, application of a sclerostin neutralizing antibody has also been shown to enhance metaphyseal bone healing in rats [78].

#### 4.1.3. Bone Morphogenic Proteins

To achieve enhanced osteogenesis, osteoconductive scaffolds can be used in combination with osteoinductive agents. Such agents were first described in the 1960s, when Urist showed that acellular, devitalized and decalcified bone matrix could induce bone formation in ectopic tissue [46]. Today, the best studied group of growth factors used in the treatment of skeletal defects are bone BMPs. BMPs belong to the TGF-β superfamily and are known to promote MSC differentiation into chondroblasts or osteoblasts and facilitate bone formation [79]. BMPs act via the Smad dependent canonical pathway or the Smad independent non-canonical pathways including the mitogen-activated protein kinase (MAPK) signaling cascade [80]. Ligand binding to the type I and II serine/threonine kinase receptor containing heterotetrametric receptor complex leads to phosphorylation of Smad1/5/8 which further associates with Smad4 and translocates to the nucleus where it induces target gene transcription. Downstream targets of BMP-2 for example include Runx2, Dlx5 and osterix [81,82,83] (Figure 4).

Recombinant human BMP-2 (rhBMP-2) and rhBMP-7 were approved by the FDA for specific clinical indications. The use BMP-2 was shown beneficial in the treatment of acute tibial fractures [84]. Augmentation of cancellous allograft with rhBMP-2 achieved similar healing rates as iliac crest autografts in the treatment tibial shaft fractures with cortical defects [85]. In spine surgery, rhBMP-2 proved to be beneficial in cases of anterior interbody lumbar fusion and was approved by the FDA in 2002 [86,87]. Its use, also off-label in other forms of lumbar as well as cervical fusion has increased tremendously after approval, however, significantly declined after safety concerns emerged in 2008 [88]. Higher rates of adverse effects like implant displacement, subsidence and infection for anterior cervical fusion as well as radiculitis, osteolysis and ectopic bone formation for posterior lumbar body fusion were observed [89,90]. Further, even acute life-threatening complications requiring intubation or tracheotomy due to cervical swelling and consecutive airway compression where described for the use rhBMP-2 [89]. In the treatment of complex tibial plateau fractures, an increased risk of heterotopic ossification was described for the use of rhBMP-2 [91].

Independent of their potential adverse effects, BMPs are still considered potent inductors of bone formation and researchers are trying to adapt these proteins in order to make their use save. Since efficacy of BMP-2 was shown to be dose dependent, the described side effects may, at least partly, be explained by relatively high concentrations used [84]. The right dosage balancing induction of bone formation and reduction of adverse effects is jet to be confirmed by clinical trials [92]. Ectopic bone formation and major complications such as spinal cord compression may be reduced by minimizing perifocal leakage and optimizing positioning of BMP-2 containing material [93,94]. Addition of bisphosphonates was intended to reduce osteoclast activity, possibly causing osteolysis and subsidence. However, the risk for heterotopic ossification was increased [95]. Adverse BMP-2 effects such as swelling, seroma formation and wound complications may be attributed to its proinflammatory properties [96,97]. A study from 2013 could demonstrate reduced edema and inflammatory response with the administration of dexamethasone during rodent spinal surgery [98]. Due to the well described negative effects of glucocorticoids on bone formation, their positive effects in bone healing must be questioned until proven otherwise. More advanced approaches to reduce inflammation were demonstrated by Gleaser et al. They could demonstrate a reduction in local inflammation with even higher bone volume and reduced trabecular spacing after adding NEMO binding domain peptide (NBD) to BMP-2. NBD is an inhibitor of NF-κB mediated inflammation and was investigated in a rat model of spinal fusion. Local edema, mononuclear cell infiltration and the expression of inflammatory markers was reduced when BMP-2 was used with NBD compared to BMP-2 alone [99].

#### 4.1.4. Platelet Derived Growth Factor

As the strive for iliac crest autograft substitutes continues, PDGF was found to be a promising candidate to promote bone regeneration. PDGF is found in the early fracture hematoma where it is expressed by endothelial and mesenchymal cells following the primary insult. In the course of fracture healing, it is also expressed by osteoblasts, chondrocytes and even osteoclasts [100]. PDGF acts as a chemotactic as well as mitogenic agent on MSCs, promoting osteoblast and chondrocyte differentiation. Additionally, PDGF upregulates VEGF which enhances angiogenesis [101]. The chemotactic potential of PDGF on MSC was shown to be dose dependent and superior to the chemotactic effects of other cytokines [102]. Further, the PDGF-BB homodimer was found to exhibit superior chemotactic and proliferative effects on MSC compared to other PDGF isoforms and different growth factors [103]. PDGF signals through the PDGF receptors α and β, which may be present as homo- or heterodimers and signal via a tyrosine kinase. PDGF-BB has the potential to activate either isoform of the dimeric receptor [104].

In terms of fracture healing PDGF must be used with caution. Imatinib, a monoclonal antibody inhibiting tyrosine kinase activity, was shown to promote osteoblast differentiation by inhibiting PDGF receptor β (PDGFRβ) signaling [105]. Further it was demonstrated that PDGFRβ signaling induces MSC proliferation and migration but inhibits osteogenic differentiation [106]. A recent study by Wang et al. found an increased expression of PDGFRβ early after fracture within the thickened periosteum as well as the fracture callus. Those finding were associated with increased proliferation and decreased apoptosis of PDGF receptor expressing periosteum-derived progenitor cells (PDC) upon PDGF stimulation. Surprisingly though, PDGF inhibited BMP-2 induced osteogenesis via PDGFRβ signaling. ERK1/2 MAPK and PI3K/AKT were identified as two downstream signaling pathways exerting the suppressive PDGF/PDGFRβ effects on BMP-2 induced PDCs [107]. Obviously PDGF exerts a strong proliferative effect on osteoblasts while inhibiting their differentiation.

Preclinical trials on the use of PDGF overexpressing hematopoietic stem cells in mice demonstrated increased trabecular bone formation and trabecular connectivity as well as decreased cortical porosity, adding up to a 45% increase in bone strength with low dose treatment [108]. Those effects were attributed to enhanced proliferation of MSC and angiogenesis. The therapeutic effect though, appears highly dependent on adequate dosing, since higher concentrations of PDGF induced largely unmineralized bone. In clinical applications, PDGF is established in ankle and hindfoot arthrodesis. Recombinant human PDGF-BB homodimer (rhPDGF-BB) in combination with an osteoinductive tricalcium phosphate (TCP)-collagen matrix was shown to be a safe and effective alternative to autografts in terms of clinical, functional and radiologic outcomes, eliminating harvesting site morbidity [109]. Similar results were reported for the use of an injectable TCP-collagen matrix in combination with rhPDGF-BB [110]. FDA approval for rhPDGF-BB/TCP-collagen in ankle and/or hindfoot fusion was granted in 2015 and extended to an injectable form in 2018.

#### 4.1.5. Fibroblast Growth Factor

FGFs describe a large family of 22 ligands binding to four receptor tyrosine kinases essential for proper tissue development and metabolism [111]. The important role of FGFs and their corresponding receptors in osteogenesis is well established by various studies, but their function in fracture repair is incompletely understood [112]. FGF-2 is probably the most studied FGF in terms of bone metabolism. FGF-2 has well established anabolic as well as catabolic function. While low concentrations favor osteoclastic bone resorption, high concentrations are osteoanabolic [113]. Intravenous application of FGF-2 was found to stimulate osteoblast proliferation and new bone formation in rats [114]. In a rat fibula fracture model, local FGF-2 injection immediately after fracture could increase the mineral content and callus volume resulting in superior mechanical properties [115]. Those anabolic effects were supported by Nakamura et al. in 1998 and extended by the observation of an increased number of osteoclasts upon FGF-2. The authors attribute the positive effect of FGF-2 on bone healing to its induction of bone remodeling, rather than purely anabolic effects [116]. Kawaguchi et al. conducted a trial on the use of recombinant human FGF-2 (rhFGF-2) in patients with high tibial closed wedge osteotomies due to medial compartment osteoarthritis. Patients received a total dosage of 200, 400 or 800 µg of rhFGF-2 in a biodegradable hydrogel carrier. The authors found a dose dependent positive effect on radiographic union, accelerated union and better clinical outcomes. No adverse effects were observed [117]. Similarly, a later study by the same group showed beneficial effects on radiographic union of local rhFGF-2 application in nailed tibial fractures [113]

FGF-2 interacts closely with other mediators involved in bone metabolism described above. Xiao et al. described a low molecular weight isoform of FGF-2 to carry out the anabolic FGF-2 function by modulating Wnt signaling [118]. Overexpression of this isoform in the osteoblastic lineage resulted in accelerated fracture healing due to faster cartilage formation, bone union and callus remodeling in a tibial mouse fracture model [119]. Interestingly, FGF-2 and BMP-2 were shown to act synergistically in bone formation. Deficiency in FGF-2 leads to reduced expression and function of BMP-2 [120]. Additionally, the FGF-2 enhanced enchondral ossification potential of periosteal cells is driven by an increased production of BMP-2 [120,121]. PTH has also been described do exert its effects on bone metabolism, at least in part, through FGF-2 by upregulating FGF-2 and FGFR mRNA expression in osteoblastic cells in vitro [122]. The same group could show an impaired osteoanabolic response to PTH in FGF-2-deficient mice, possibly due to impaired Wnt/β-catenin signaling that was significantly decreased in bone marrow stromal cells during osteoblast differentiation [123,124].

Most research has been focused on enhancing the anabolic effects of FGFs, while blockage of inhibitory effects of FGFs could be just as valuable. Overexpression of FGF-23 has been shown to inhibit osteoblast differentiation and matrix mineralization in vitro [125]. For osteocytes PTHR signaling was shown to increase FGF-23 expression [126].

Besides FGFs itself, FGF receptors (FGFR) and their downstream signaling have moved into focus of research. Impaired FGFR3 signaling was found to be associated with defects in skeletogenesis. While activating mutations cause small statue, inactivating mutations result in tall statue [127,128]. In a rat femur fracture model, Rundle et al. could show a differential expression of the FGFR genes. While FGFR1 and 2 are expressed throughout fracture repair, FGFR3 expression is seen in chondrocytes and osteoblasts from day 10 on and persisted during enchondral bone formation [129]. Paradoxically, deficiency as well as overactivation of FGFR3 signaling is associated with osteopenia [130,131]. A more recent study demonstrated accelerated fracture healing in FGFR3-deficient mice. This effect was attributed to increased cartilage and fracture callus formation plus a more a rapid enchondral ossification. The authors postulate that blockage of FGFR3 signaling could be beneficial in the treatment of patients with impaired fracture healing [132]. However, an isolated deletion of FGFR3 signaling in osteoclasts leads to impaired callus remodeling in fracture repair due to reduced osteoclast bone resorption. This study could also demonstrate a reduced, but not entirely abolished, response to FGF-2 induced osteoclastic bone resorption in FGFR3 deficient osteoclast [133].

### 4.2. Novel Bone Grafts

As described above, research in the past decades has identified various osteoanabolic agents which are currently under further investigation and some of which are already applied clinically. While systemic diseases such as osteoporosis increase the risk for impaired bone regeneration, young and healthy individuals may also experience non-unions, pointing towards a local pathology. From a clinical point of view, local therapy is therefore preferable as it additionally minimizes the risk for possible side effects. The locally applied agent’s potential to promote bone formation will, however, depend on a suitable scaffold, allowing adequate cell migration, proliferation, and differentiation as well as matrix production and degradation. While currently used natural bone grafts have been discussed above, an increasing number of artificial bone grafts may be applied in the treatment of fracture non-union. Importantly, artificial bone grafts, as an alternative to natural auto- and allografts, are not associated with donor site morbidity or the possibility of immunogenic complications. Additionally, they exist, at least theoretically, at unlimited availability. The ideal bone graft shows osteogenic, osteoinductive and osteoconductive properties, is perfectly biocompatible with no immunogenic reaction, biodegradable without harmful byproducts, allows or even induces neovascularization, and provides a certain degree of mechanical stability.

#### 4.2.1. Calcium-Based Bone Grafts

Calcium based bone grafts include calcium phosphate ceramics, calcium phosphate cements and calcium sulfate. Calcium phosphate-based biomaterials were shown to have osteoconductive properties that are attributed to their microporous structure [134]. Calcium sulfates miss a porous microstructure which implies a lack of osteoconductivity. Furthermore, calcium sulfates are associated with early resorption within 4–8 weeks and inferior mechanical strength which limits their use to small bone defects or in combination with rigid fixation. [135]. Additionally, calcium sulfates have been associated with prolonged serous wound drainage as calcium sulfate is degraded [136].

Calcium phosphates such as hydroxyapatite (HA) and TCP have been widely investigated and used in bone defects. In healthy bone, HA is produced by osteoblasts and represents the major anorganic component of the bone matrix. The biocompatibility of HA bone grafts is thought to result from its structural similarity to natural bone matrix [137]. Scaffolds made from HA show good mechanical strength but are resorbed slowly, adding to fracture risk of new bone. TCP in contrast shows weak mechanical properties but is biodegraded quickly [138]. Biphasic ceramics composed of HA and TCP allow to achieve the desired mechanical and biodegradable characteristics depended on the HA/TCP ratio [139]. Calcium phosphate cements (CPC), in contrast to ceramics, are composed of a combination of calcium phosphates which are soluble and self-harden under in vivo conditions [140]. Their solubility and easy processability make them convenient for filling bone defects [141]. CPC exert osteoconductive properties and show adequate biodegradability, and bone healing potential can be enhanced by adding osteoinductive growth factors [142].

Nowadays however, modern scaffolds are generally produced from a biodegradable polymer and the ceramic component. Advanced biodegradable polymers such as poly(lactic-co-glycolic acid) (PLGA) and poly(L-lactic acid) (PLLA) in the combination with a ceramic were able to overcome limitations of conventional ceramic bone substitutes [143,144]. Additionally, new methods of fabricating those scaffolds including the use of gas forming and particulate leaching (GF/PL) instead of solvent casting and particulate leaching (SC/PL) could even enhance their bone regeneration efficacy, possibly due to better exposure of HA to osteogenic cells [145]. Both polymer/HA as well as CPC scaffolds have been successfully used as a delivery system for pluripotent stem cells in animal trials [144,146].

#### 4.2.2. Bioactive Glass

Bioactive glass describes silicate-based ceramics with bioactive properties. It was first described in 1971 and recognized for its ability to bind host bone [147]. Besides this characteristic, bioglass exhibits osteoconductive properties due to its porous nature and is resorbed quickly with only little inflammatory response [148]. Besides their positive effects on osteoinduction, they were also described to have antimicrobial activity. During bioglass degradation, dissolving salts create a local increase in pH and osmotic pressure which was reported to be bacteriocidal [149,150]. Most interestingly, in the field of orthopedic surgery, in vitro studies demonstrated bioactive glass S53P4 to suppress Staphylococcus aureus biofilm formation on titanium plates. Such effect could be of tremendous importance in aseptic or septic exchange arthroplasty and the treatment of osteomyelitis [151]. In this respect, Drago et al. reported healing in 24 out of 27 patients with osteomyelitis of long bones after surgical debridement and defect filling with bioactive glass [152]. Today, bioactive glass has been approved for several indications in North America and Europe and is considered a valuable bone substitute for long bone infections [153,154]. Further interest has risen in the use of therapeutic ions which can be released from bioactive glass and display antibacterial, anti-inflammatory as well as osteogenic and angiogenic properties. The list of ions includes silver, lithium, fluoride, calcium, strontium, manganese, magnesium, zinc, copper, cobalt, cerium, gallium, boron, iron, europium, silicon, phosphate and niobium. Despite their encouraging characteristics, the right dosing preventing unwanted cytotoxicity and controlled release from the delivery vehicle remains challenging [154].

#### 4.2.3. Organic Bone Grafts

Organic bone grafts are nanocomposites of mainly type I collagen mimicking the natural collagen scaffold. Mineralization is mainly attributed to non-collagenous proteins (NCPs) and collagen is considered to provide a passive mechanical support for mineralization [155]. However, several studies also demonstrated that collagen actively controls mineralization by synergizing with inhibitors of hydroxyapatite nucleation [156,157]. To enhance osteoconductive characteristics, collagen scaffolds are generally combined with calcium phosphates, growth factors or even stem cells [144,158,159]. Besides natural collagen polymers, synthetic resorbable polymers have been introduced. Those include PLLA and PLGA polymers which can be used alone or as extenders for auto- and allografts, and in combination with ceramics or stem cells [42,143,144].

#### 4.2.4. Innovative Biomaterials

As new technologies are developed and, in several cases, have revolutionized whole industries, the field of medicine is no exception. Huge progress has been made in the field of tissue engineering. One the one hand, advances in biology allow the production of bioinspired tissue which resembles the properties of native tissue. On the other hand, modern technologies such as 3D printing enable scientists to produce customized implants with high structural complexity on the nanoscopic level. In the case of non-unions with impaired local biology combined with the challenging tissue characteristics of bone and the often-large defects, patients could profit tremendously from these novel approaches, and current expectations are high. Up to now, 3D printed scaffolds are used in orthopedic trauma surgery to improve pre-operative planning with personalized anatomic models, and to overcome complex anatomic differences or defects which cannot be met with conventional implants [160]. 3D printed implants are successfully used in maxillofacial and pelvic reconstructive surgery [161,162], however, currently used implants have no or only limited biologic activity. This is surprising, as 3D printed scaffolds were experimentally shown to be able to exert osteoconductive effects. Therefore, future research will focus on how to enhance the biologic activity of these implants by adding osteoinductive and osteogenic properties by integration of growth factors or stem cells. Furthermore, their function as a vehicle for drug delivery must be addressed [163,164].

Despite these limitations, recent advances in biomaterials are promising. Bioinspired composite matrices composed of a crystalline, rod-shaped nanoHA core and an amorphous silica sheath (Si-nHA) were shown to have good biocompatibility, osteogenic differentiation, vascularization and bone regeneration potential. In a critical size femoral defect rat model, this matrix resulted in complete bridging and union of the bone gap [165]. Turner et al. demonstrated a method of biomineralization of HA, creating the favorable biogenic HA instead of the widely used biomimetic HA [166]. Moreover, biomimetic vascularized organoids demonstrating fundamental tissue functions can now be fabricated [167].

Since tissue, including bone repair, is a precisely regulated process with a wide diversity of growth factors and cell types exerting their function at a different time, sequential stimulation is attracting attention. Incorporation of growth factors and even cells into implants and their release at a desired point of time promises a more specific and hopefully effective support in tissue regeneration. Electrochemical-, pH- and UV triggered agent release were demonstrated [168,169,170]. For in vivo use even more relevant, He et al. could demonstrate inflammation-induced and rate-dependent release of indomethacin conjugated to a porous hydrogel coating, holding great potential for the prophylaxis of heterotopic ossification [171].

One last aspect of implants is that of degradability. Patients with implant-associated pain or foreign body feeling may require an additional surgical intervention for implant removal, with the potential for peri-operative complications [172]. Therefore, implants providing mechanical support after fracture fixation but subsequent degradation after fracture union are most desirable. As described above, biodegradable polymers are currently available, but only suitable for low-load bearing fracture sites [42]. As of now, magnesium-based orthopedic implants show promising results, since they combine biodegradability with appropriate mechanical strength and additionally promote the formation of new bone [173].

## 5. Conclusions

Despite tremendous scientific and clinical effort, impaired bone healing still represents a complex and challenging complication following a fracture. A detailed case history, state-of-the-art diagnostics, and individualized treatment concepts are crucial for optimal patient outcome. Scientific advances in deeper understanding the molecular processes governing fracture healing have resulted in the identification of key mediators which can potentially be targeted to promote bone regeneration. The improvement of currently available bone substitute materials and the development of innovative biomaterials have significantly contributed to expand available treatment options. Therefore, further research and identification of novel therapeutic approaches with adequate safety profiles fulfills an essential clinical need, to promote bone regeneration and restore bone defects in patients suffering from non-unions.

## Figures and Tables

**Table 1:** Summary of risk factors for impaired bone regeneration (modified according to [8]).

Patient-Related Cause	Patient-Unrelated Cause
Age Gender (Male)	Open fracture (initial bone loss and soft tissue injury) Displaced fracture
Smoking ^1^ Alcohol ^1^	Wedge and multi-fragmentary fracture pattern Injury location (tibia)
High Body mass index ^1^ Malnutrition ^1^ (Calcium, Vitamin D, etc.)	Soft tissue damage - vascularisation ^2^ Open reduction - quality of primary osteosynthesis ^2^
Osteoporosis ^2^ Diabetes ^2^	Compartment syndrome ^2^ Presence of fracture gap post-surgery ^2^
Peripheral vascular disease ^2^ Chronic inflammatory disease ^2^	Poor mechanical stability by initial implant ^2^
Medication ^2^ (Insulin, Chemotherapeutics, Steroids, Antibiotics, Anticoagulants, NSAIDs, Opiates)	
Renal insufficiency Radio therapy	

^1^ Improvable, ^2^ Potetially improvable.

## Abbreviations

BMD	Bone mineral density
BMP	Bone morphogenic proteins
CPC	Calcium phosphate cements
DBM	Demineralized bone matrix
Dlx5	Distal-less homebox 5
ESTROT	European Society of Tissue Regeneration in Orthopedics and Traumatology
ESWT	Extracorporal shock wave therapy
FDA	Food and Drug Administration
FGF	Fibroblast growth factor
FGFR	FGF receptors
Fz	Frizzled receptor
GF/PL	Gas forming and particulate leaching
HA	Hydroxyapatite
IGF	Insulin like growth factor
IL	Interleukin
LIPUS	Low-intensity pulsed ultrasound
LRP5/6	LDL receptor-related protein
MAPK	Mitogen-activated protein kinase
MDSC	Muscle-derived stomal cell
MSC	Mesenchymal stem cells
NBP	NEMO binding domain peptide
NCPs	Non-collagenous proteins
PDC	Periosteum-derived progenitor cells
PDGF	Platelet derived growth factor
PDGFRβ	PDGF receptor β
PLGA	Poly(lactic-co-glycolic acid)
PLLA	Poly(l-lactic acid)
PTH	Parathyroid hormone
PTHrP	Parathyroid hormone related protein
rhBMP-2	Recombinant human BMP-2
rhFGF-2	Recombinant human FGF-2
rhPDGF-BB	Recombinant human PDGF-BB homodimer
RIA	Reamer-Irrigator-Aspirator System
Runx2	Runt-related transcription factor 2
SC/PL	Solvent casting and particulate leaching
TCP	Tricalcium phosphate
TGF-β	Transforming growth factor-β
TNF-α	Tumor necrosis factor-α

## References

[1] Zura R., Xiong Z., Einhorn T., Watson J.T., Ostrum R.F., Prayson M.J., Della Rocca G.J., Metha S., McKinley  T., Wang Z. (2016). Epidemiology of fracture nonunion in 18 human bones. JAMA Surgery.

[2] Antonova E., Le T.K., Burge R., Mershon J. (2013). Tibia shaft fractures: Costly burden of nonunions. BMC Musculoskelet. Disord..

[3] Rupp M., Biehl C., Budak M., Thormann U., Heiss C., Alt V. (2018). Diaphyseal long bone nonunions — types, aetiology, economics, and treatment recommendations. Int. Orthop..

[4] Schmidmaier G., Moghaddam A. (2015). Pseudarthrosen langer Röhrenknochen. Z. Orthop. Unfall..

[5] Calori G., Mazza E.L., Mazzola S., Colombo A., Giardina F., Romanò F., Colombo M. (2017). Non-unions. Clin. Cases Miner. Bone Metab..

[6] Weber B., Čech O. (1973). Pseudarthrosen: Pathophysiologie, Biomechanik, Therapie, Ergebnisse.

[7] Einhorn T.A. (1998). The cell and molecular biology of fracture healing. Clin. Orthop. Relat. Res..

[8] Andrzejowski P., Giannoudis P. (2019). V The “diamond concept” for long bone non-union management. J. Orthop. Traumatol..

[9] McKibbin B. (1978). The biology of fracture healing in long bones. J. Bone Joint Surg. Br..

[10] Harwood P.J., Ferguson D.O. (2015). (ii) An update on fracture healing and non-union. Orthop. Trauma.

[11] Kolar P., Schmidt-Bleek K., Schell H., Gaber T., Toben D., Schmidmaier G., Perka C., Buttgereit F., Duda G.N. (2010). The early fracture hematoma and its potential role in fracture healing. Tissue Eng. Part B Rev..

[12] Grundnes O., Reikerås O. (1993). the Importance of the Hematoma for Fracture Healing in Rats. Acta Orthop..

[13] Mizuno K., Mineo K., Tachibana T., Sumi M., Matsubara T., Hirohata K. (1990). The osteogenetic potential of fracture haematoma. Subperiosteal and intramuscular transplantation of the haematoma. J. Bone Joint Surg. Br..

[14] Young R.W. (1962). Cell proliferation and specialization during endochondral osteogenesis in young rats. J. Cell Biol..

[15] Bielby R., Jones E., McGonagle D. (2007). The role of mesenchymal stem cells in maintenance and repair of bone. Injury.

[16] Oe K., Miwa M., Sakai Y., Lee S.Y., Kuroda R., Kurosaka M. (2007). An in vitro study demonstrating that haematomas found at the site of human fractures contain progenitor cells with multilineage capacity. J. Bone Jt. Surg. Ser. B.

[17] Opal S.M. (2000). Phylogenetic and functional relationships between coagulation and the innate immune response. Crit. Care Med..

[18] Einhorn T.A., Majeska R.J., Rush E.B., Levine P.M., Horowitz M.C. (1995). The expression of cytokine activity by fracture callus. J. Bone Miner. Res..

[19] Schmidt-Bleek K., Schell H., Lienau J., Schulz N., Hoff P., Pfaff M., Schmidt G., Martin C., Perka C., Buttgereit F. (2014). Initial immune reaction and angiogenesis in bone healing. J. Tissue Eng. Regen. Med..

[20] Yang X., Ricciardi B.F., Hernandez-Soria A., Shi Y., Pleshko Camacho N., Bostrom M.P.G. (2007). Callus mineralization and maturation are delayed during fracture healing in interleukin-6 knockout mice. Bone.

[21] Wallace A., Cooney T.E., Englund R., Lubahn J.D. (2011). Effects of interleukin-6 ablation on fracture healing in mice. J. Orthop. Res..

[22] Gerstenfeld L.C., Cho T.J., Kon T., Aizawa T., Tsay A., Fitch J., Barnes G.L., Graves D.T., Einhorn T.A. (2003). Impaired fracture healing in the absence of TNF-α signaling: The role of TNF-α in endochondral cartilage resorption. J. Bone Miner. Res..

[23] Glass G.E., Chan J.K., Freidin A., Feldmann M., Horwood N.J., Nanchahal J. (2011). TNF-α promotes fracture repair by augmenting the recruitment and differentiation of muscle-derived stromal cells. Proc. Natl. Acad. Sci. USA.

[24] Brighton C.T., Hunt R.M. (1991). Early histological and ultrastructural changes in medullary fracture callus. J. Bone Jt. Surg. Ser. A.

[25] Lieberman J.R., Daluiski A., Einhorn T.A. (2002). The role of growth factors in the repair of bone biology and clinical applications. J. Bone Jt. Surg. Ser. A.

[26] Ford J.L., Robinson D.E., Scammell B.E. (2003). The fate of soft callus chondrocytes during long bone fracture repair. J. Orthop. Res..

[27] Manolagas S.C. (2000). Birth and death of bone cells: Basic regulatory mechanisms and implications for the pathogenesis and treatment of osteoporosis. Endocr. Rev..

[28] Schenk R., Willenegger H. (1964). [On the histology of primary bone healing]. Langenbecks Arch. Klin. Chir. Ver. Dtsch. Z. Chir..

[29] Gerstenfeld L.C., Alkhiary Y.M., Krall E.A., Nicholls F.H., Stapleton S.N., Fitch J.L., Bauer M., Kayal R., Graves D.T., Jepsen K.J. (2006). Three-dimensional reconstruction of fracture callus morphogenesis. J. Histochem. Cytochem..

[30] Marsell R., Einhorn T.A. (2011). The biology of fracture healing. Injury.

[31] Thompson Z., Miclau T., Hu D., Helms J.A. (2002). A model for intramembranous ossification during fracture healing. J. Orthop. Res..

[32] Calori G.M., Phillips M., Jeetle S., Tagliabue L., Giannoudis P.V. (2008). Classification of non-union: Need for a new scoring system?. Injury.

[33] Gebauer D., Mayr E., Orthner E., Ryaby J.P. (2005). Low-intensity pulsed ultrasound: Effects on nonunions. Ultrasound Med. Biol..

[34] Schoellner C., Rompe J.-D., Decking J., Heine J. (2002). Die hochenergetische extrakorporale Stoßwellentherapie (ESWT) bei Pseudarthrose. Orthopade.

[35] Lou S., Lv H., Li Z., Zhang L., Tang P. (2017). The effects of low-intensity pulsed ultrasound on fresh fracture. Medicine (Baltimore).

[36] Leighton R., Watson J.T., Giannoudis P., Papakostidis C., Harrison A., Steen R.G. (2017). Healing of fracture nonunions treated with low-intensity pulsed ultrasound (LIPUS): A systematic review and meta-analysis. Injury.

[37] Schlickewei W., Schlickewei C. (2007). The Use of Bone Substitutes in the Treatment of Bone Defects – the Clinical View and History. Macromol. Symp..

[38] Cox G., McGonagle D., Boxall S.A., Buckley C.T., Jones E., Giannoudis P.V. (2011). The use of the reamer-irrigator-aspirator to harvest mesenchymal stem cells. J. Bone Joint Surg. Br..

[39] Madison R.D., Nowotarski P.J. (2019). The Reamer-Irrigator-Aspirator in Nonunion Surgery. Orthop. Clin. N. Am..

[40] Calori G.M., Colombo M., Mazza E.L., Mazzola S., Malagoli E., Mineo G.V. (2014). Incidence of donor site morbidity following harvesting from iliac crest or RIA graft. Injury.

[41] Schmidmaier G., Herrmann S., Green J., Weber T., Scharfenberger A., Haas N.P., Wildemann B. (2006). Quantitative assessment of growth factors in reaming aspirate, iliac crest, and platelet preparation. Bone.

[42] Laurencin C., Khan Y., El-Amin S.F. (2006). Bone graft substitutes. Expert Rev. Med. Devices.

[43] Herrera M., Chapman C.B., Roh M., Strauch R.J., Rosenwasser M.P. (1999). Treatment of unstable distal radius fractures with cancellous allograft and external fixation. J. Hand Surg. Am..

[44] Lasanianos N., Mouzopoulos G., Garnavos C. (2008). The use of freeze-dried cancelous allograft in the management of impacted tibial plateau fractures. Injury.

[45] Flierl M.A., Smith W.R., Mauffrey C., Irgit K., Williams A.E., Ross E., Peacher G., Hak D.J., Stahel P.F. (2013). Outcomes and complication rates of different bone grafting modalities in long bone fracture nonunions: A retrospective cohort study in 182 patients. J. Orthop. Surg. Res..

[46] Urist M.R. (1965). Bone: Formation by autoinduction. Science.

[47] Hierholzer C., Sama D., Toro J.B., Peterson M., Helfet D.L. (2006). Plate Fixation of Ununited Humeral Shaft Fractures. J. Bone Jt. Surg..

[48] Peterson R.S., Andhare R.A., Rousche K.T., Knudson W., Wang W., Grossfield J.B., Thomas R.O., Hollingsworth R.E., Knudson C.B. (2004). CD44 modulates Smad1 activation in the BMP-7 signaling pathway. J. Cell Biol..

[49] Wildemann B., Burkhardt N., Luebberstedt M., Vordemvenne T., Schmidmaier G. (2007). Proliferating and differentiating effects of three different growth factors on pluripotent mesenchymal cells and osteoblast like cells. J. Orthop. Surg. Res..

[50] Masquelet A.C., Fitoussi F., Begue T., Muller G.P. (2000). Reconstruction of the long bones by the induced membrane and spongy autograft. Annales de Chirurgie Plastique et Esthetique.

[51] Morelli I., Drago L., George D.A., Gallazzi E., Scarponi S., Romanò C.L. (2016). Masquelet technique: Myth or reality? A systematic review and meta-analysis. Injury.

[52] Silva B.C., Costa A.G., Cusano N.E., Kousteni S., Bilezikian J.P. (2011). Catabolic and anabolic actions of parathyroid hormone on the skeleton. J. Endocrinol. Invest..

[53] Dobnig H., Turner R.T. (1997). The effects of programmed administration of human parathyroid hormone fragment (1–34) on bone histomorphometry and serum chemistry in rats. Endocrinology.

[54] Neer R.M., Arnaud C.D., Zanchetta J.R., Prince R., Gaich G.A., Reginster J.-Y., Hodsman A.B., Eriksen E.F., Ish-Shalom S., Genant H.K. (2001). Effect of Parathyroid Hormone (1-34) on Fractures and Bone Mineral Density in Postmenopausal Women with Osteoporosis. N. Engl. J. Med..

[55] Greenspan S.L., Bone H.G., Ettinger M.P., Hanley D.A., Lindsay R., Zanchetta J.R., Blosch C.M., Mathisen A.L., Morris S.A., Marriott T.B. (2007). Effect of Recombinant Human Parathyroid Hormone (1-84) on Vertebral Fracture and Bone Mineral Density in Postmenopausal Women with Osteoporosis. Ann. Intern. Med..

[56] Miller P.D., Hattersley G., Riis B.J., Williams G.C., Lau E., Russo L.A., Alexandersen P., Zerbini C.A.F., Hu M., Harris A.G. (2016). Effect of Abaloparatide vs Placebo on New Vertebral Fractures in Postmenopausal Women With Osteoporosis. JAMA.

[57] Miller P.D., Hattersley G., Lau E., Fitzpatrick L.A., Harris A.G., Williams G.C., Hu M.-Y., Riis B.J., Russo L., Christiansen C. (2019). Bone mineral density response rates are greater in patients treated with abaloparatide compared with those treated with placebo or teriparatide: Results from the ACTIVE phase 3 trial. Bone.

[58] Jilka R.L. (2007). Molecular and cellular mechanisms of the anabolic effect of intermittent PTH. Bone.

[59] Nozaka K., Miyakoshi N., Kasukawa Y., Maekawa S., Noguchi H., Shimada Y. (2008). Intermittent administration of human parathyroid hormone enhances bone formation and union at the site of cancellous bone osteotomy in normal and ovariectomized rats. Bone.

[60] Aspenberg P., Genant H.K., Johansson T., Nino A.J., See K., Krohn K., García-Hernández P.A., Recknor C.P., Einhorn T.A., Dalsky G.P. (2010). Teriparatide for acceleration of fracture repair in humans: A prospective, randomized, double-blind study of 102 postmenopausal women with distal radial fractures. J. Bone Miner. Res..

[61] Peichl P., Holzer L.A., Maier R., Holzer G. (2011). Parathyroid hormone 1-84 accelerates fracture-healing in pubic bones of elderly osteoporotic women. J. Bone Jt. Surg. Ser. A.

[62] Bukata S.V., Puzas J.E. (2010). Orthopedic Uses of Teriparatide. Curr. Osteoporos. Rep..

[63] Zati A., Sarti D., Malaguti M.C., Pratelli L., Rohe M.E., Krege J.H. (2011). Teriparatide in the treatment of a loose hip prosthesis. J. Rheumatol..

[64] Jiang Y., Zhao J.J., Mitlak B.H., Wang O., Genant H.K., Eriksen E.F. (2003). Recombinant Human Parathyroid Hormone (1-34) [Teriparatide] Improves Both Cortical and Cancellous Bone Structure. J. Bone Miner. Res..

[65] Ogura K., Iimura T., Makino Y., Sugie-Oya A., Takakura A., Takao-Kawabata R., Ishizuya T., Moriyama K., Yamaguchi A. (2016). Short-term intermittent administration of parathyroid hormone facilitates osteogenesis by different mechanisms in cancellous and cortical bone. Bone Rep..

[66] Bellido T., Ali A.A., Gubrij I., Plotkin L.I., Fu Q., O’Brien C.A., Manolagas S.C., Jilka R.L. (2005). Chronic elevation of parathyroid hormone in mice reduces expression of sclerostin by osteocytes: A novel mechanism for hormonal control of osteoblastogenesis. Endocrinology.

[67] Ten Dijke P., Krause C., de Gorter D.J., Löwik C.W., van Bezooijen R.L. (2008). Osteocyte-Derived Sclerostin Inhibits Bone Formation: Its Role in Bone Morphogenetic Protein and Wnt Signaling. J. Bone Jt. Surgery-Am. Vol..

[68] Delgado-Calle J., Tu X., Pacheco-Costa R., McAndrews K., Edwards R., Pellegrini G.G., Kuhlenschmidt K., Olivos N., Robling A., Peacock M. (2017). Control of Bone Anabolism in Response to Mechanical Loading and PTH by Distinct Mechanisms Downstream of the PTH Receptor. J. Bone Miner. Res..

[69] Bennett C.N., Longo K.A., Wright W.S., Suva L.J., Lane T.F., Hankenson K.D., MacDougald O.A. (2005). Regulation of osteoblastogenesis and bone mass by Wnt10b. Proc. Natl. Acad. Sci. USA.

[70] Cawthorn W.P., Bree A.J., Yao Y., Du B., Hemati N., Martinez-Santibañez G., MacDougald O.A. (2012). Wnt6, Wnt10a and Wnt10b inhibit adipogenesis and stimulate osteoblastogenesis through a β-catenin-dependent mechanism. Bone.

[71] Chen Y., Whetstone H.C., Lin A.C., Nadesan P., Wei Q., Poon R., Alman B.A. (2007). Beta-catenin signaling plays a disparate role in different phases of fracture repair: Implications for therapy to improve bone healing. PLoS Med..

[72] Kakar S., Einhorn T.A., Vora S., Miara L.J., Hon G., Wigner N.A., Toben D., Jacobsen K.A., Al-Sebaei M.O., Song M. (2007). Enhanced chondrogenesis and Wnt signaling in PTH-treated fractures. J. Bone Miner. Res..

[73] Liu T.M., Lee E.H. (2013). Transcriptional regulatory cascades in Runx2-dependent bone development. Tissue Eng. Part B Rev..

[74] Wojtowicz A.M., Templeman K.L., Hutmacher D.W., Guldberg R.E., García A.J. (2010). Runx2 overexpression in bone marrow stromal cells accelerates bone formation in critical-sized femoral defects. Tissue Eng. Part A.

[75] Brunkow M.E., Gardner J.C., Van Ness J., Paeper B.W., Kovacevich B.R., Proll S., Skonier J.E., Zhao L., Sabo P.J., Fu Y. (2001). Bone dysplasia sclerosteosis results from loss of the SOST gene product, a novel cystine knot-containing protein. Am. J. Hum. Genet..

[76] Balemans W., Ebeling M., Patel N., Van Hul E., Olson P., Dioszegi M., Lacza C., Wuyts W., Van Den Ende J., Willems P. (2001). Increased bone density in sclerosteosis is due to the deficiency of a novel secreted protein (SOST). Hum. Mol. Genet..

[77] Cosman F., Crittenden D.B., Adachi J.D., Binkley N., Czerwinski E., Ferrari S., Hofbauer L.C., Lau E., Lewiecki E.M., Miyauchi A. (2016). Romosozumab Treatment in Postmenopausal Women with Osteoporosis. N. Engl. J. Med..

[78] Agholme F., Li X., Isaksson H., Ke H.Z., Aspenberg P. (2010). Sclerostin antibody treatment enhances metaphyseal bone healing in rats. J. Bone Miner. Res..

[79] Wozney J.M. (2002). Overview of bone morphogenetic proteins. Spine (Phila. Pa. 1976).

[80] Wang R.N., Green J., Wang Z., Deng Y., Qiao M., Peabody M., Zhang Q., Ye J., Yan Z., Denduluri S. (2014). Bone morphogenetic protein (BMP) signaling in development and human diseases. Genes Dis..

[81] Lee K.-S., Kim H.-J., Li Q.-L., Chi X.-Z., Ueta C., Komori T., Wozney J.M., Kim E.-G., Choi J.-Y., Ryoo H.-M. (2000). Runx2 Is a common target of transforming growth factor beta 1 and bone morphogenetic protein 2, and cooperation between Runx2 and Smad5 induces osteoblast-specific gene expression in the pluripotent mesenchymal precursor cell line C2C12. Mol. Cell. Biol..

[82] Miyama K., Yamada G., Yamamoto T.S., Takagi C., Miyado K., Sakai M., Ueno N., Shibuya H. (1999). A BMP-inducible gene, Dlx5, regulates osteoblast differentiation and mesoderm induction. Dev. Biol..

[83] Nakashima K., Zhou X., Kunkel G., Zhang Z., Deng J.M., Behringer R.R., De Crombrugghe B. (2002). The novel zinc finger-containing transcription factor osterix is required for osteoblast differentiation and bone formation. Cell.

[84] Govender S., Csimma C., Genant H.K., Valentin-Opran A., Amit Y., Arbel R., Aro H., Atar D., Bishay M., Börner M.G. (2002). Recombinant human bone morphogenetic protein-2 for treatment of open tibial fractures: A prospective, controlled, randomized study of four hundred and fifty patients. J. Bone Jt. Surg. Ser. A.

[85] Jones A.L., Bucholz R.W., Bosse M.J., Mirza S.K., Lyon T.R., Webb L.X., Pollak A.N., Golden J.D., Valentin-Opran A. (2006). Recombinant human BMP-2 and allograft compared with autogenous bone graft for reconstruction of diaphyseal tibial fractures with cortical defects: A randomized, controlled trial. J. Bone Jt. Surg. Ser. A.

[86] Burkus J.K., Transfeldt E.E., Kitchel S.H., Watkins R.G., Balderston R.A. (2002). Clinical and radiographic outcomes of anterior lumbar interbody fusion using recombinant human bone morphogenetic protein-2. Spine (Phila. Pa. 1976).

[87] Burkus J.K., Gornet M.F., Dickman C.A., Zdeblick T.A. (2002). Anterior lumbar interbody fusion using rhBMP-2 with tapered interbody cages. J. Spinal Disord. Tech..

[88] Martin B.I., Lurie J.D., Tosteson A.N.A., Deyo R.A., Farrokhi F.R., Mirza S.K. (2015). Use of bone morphogenetic protein among patients undergoing fusion for degenerative diagnoses in the United States, 2002 to 2012. Spine J..

[89] Carragee E.J., Hurwitz E.L., Weiner B.K. (2011). A critical review of recombinant human bone morphogenetic protein-2 trials in spinal surgery: Emerging safety concerns and lessons learned. Spine J..

[90] Tannoury C.A., An H.S. (2014). Complications with the use of bone morphogenetic protein 2 (BMP-2) in spine surgery. Spine J..

[91] Boraiah S., Paul O., Hawkes D., Wickham M., Lorich D.G. (2009). Complications of recombinant human BMP-2 for treating complex tibial plateau fractures: A preliminary report. Clin. Orthop. Relat. Res..

[92] James A.W., LaChaud G., Shen J., Asatrian G., Nguyen V., Zhang X., Ting K., Soo C. (2016). A Review of the clinical side effects of bone morphogenetic protein-2. Tissue Eng. Part B Rev..

[93] Rihn J.A., Makda J., Hong J., Patel R., Hilibrand A.S., Anderson D.G., Vaccaro A.R., Albert T.J. (2009). The use of RhBMP-2 in single-level transforaminal lumbar interbody fusion: A clinical and radiographic analysis. Eur. Spine J..

[94] Pradhan B.B., Bae H.W., Kropf M.A., Patel V.V., Zhao L., Wong P., Delamarter R.B. (2005). Leakage of rhBMP-2 from absorbable collagen sponges during use in anterior cervical discectomy and fusion quantification and follow-up of clinical and radiographic consequences. Spine J..

[95] Kim H.K.W., Aruwajoye O., Du J., Kamiya N. (2014). Local administration of bone morphogenetic protein-2 and bisphosphonate during non-weight-bearing treatment of ischemic osteonecrosis of the femoral head: An experimental investigation in immature pigs. J. Bone Jt. Surgery-Am. Vol..

[96] Robin B.N., Chaput C.D., Zeitouni S., Rahm M.D., Zerris V.A., Sampson H.W. (2010). Cytokine-mediated inflammatory reaction following posterior cervical decompression and fusion associated with recombinant human bone morphogenetic protein-2: A case study. Spine (Phila. Pa. 1976).

[97] Zara J.N., Siu R.K., Zhang X., Shen J., Ngo R., Lee M., Li W., Chiang M., Chung J., Kwak J. (2011). High doses of bone morphogenetic protein 2 induce structurally abnormal bone and inflammation In vivo. Tissue Eng. Part A.

[98] Xiong C., Daubs M.D., Montgomery S.R., Aghdasi B., Inoue H., Tian H., Suzuki A., Tan Y., Hayashi T., Ruangchainikom M. (2013). BMP-2 adverse reactions treated with human dose equivalent dexamethasone in a rodent model of soft-tissue inflammation. Spine (Phila. Pa. 1976).

[99] Glaeser J.D., Salehi K., Kanim L.E.A., Sheyn D., Napier Z., Behrens P.H., Garcia L., Cuéllar J.M., Bae H.W. (2018). Anti-Inflammatory Peptide Attenuates Edema and Promotes BMP-2-Induced Bone Formation in Spine Fusion. Tissue Eng. Part A.

[100] Andrew J.G., Hoyland J.A., Freemont A.J., Marsh D.R. (1995). Platelet-derived growth factor expression in normally healing human fractures. Bone.

[101] Hollinger J.O., Hart C.E., Hirsch S.N., Lynch S., Friedlaender G.E. (2008). Recombinant human platelet-derived growth factor: Biology and clinical applications. J. Bone Jt. Surg. Ser. A.

[102] Fiedler J., Röderer G., Günther K.-P., Brenner R.E. (2002). BMP-2, BMP-4, and PDGF-bb stimulate chemotactic migration of primary human mesenchymal progenitor cells. J. Cell. Biochem..

[103] Ozaki Y., Nishimura M., Sekiya K., Suehiro F., Kanawa M., Nikawa H., Hamada T., Kato Y. (2007). Comprehensive analysis of chemotactic factors for bone marrow mesenchymal stem cells. Stem Cells Dev..

[104] Graham S., Leonidou A., Lester M., Heliotis M., Mantalaris A., Tsiridis E. (2009). Investigating the role of PDGF as a potential drug therapy in bone formation and fracture healing. Expert Opin. Investig. Drugs.

[105] O’Sullivan S., Naot D., Callon K., Porteous F., Horne A., Wattie D., Watson M., Cornish J., Browett P., Grey A. (2007). Imatinib promotes osteoblast differentiation by inhibiting PDGFR signaling and inhibits osteoclastogenesis by both direct and stromal cell-dependent mechanisms. J. Bone Miner. Res..

[106] Tokunaga A., Oya T., Ishii Y., Motomura H., Nakamura C., Ishizawa S., Fujimori T., Nabeshima Y.I., Umezawa A., Kanamori M. (2008). PDGF receptor β is a potent regulator of mesenchymal stromal cell function. J. Bone Miner. Res..

[107] Wang X., Matthews B.G., Yu J., Novak S., Grcevic D., Sanjay A., Kalajzic I. (2019). PDGF modulates BMP2-induced osteogenesis in periosteal progenitor cells. JBMR Plus.

[108] Chen W., Baylink D.J., Brier-Jones J., Neises A., Kiroyan J.B., Rundle C.H., Lau K.-H.W., Zhang X.-B. (2015). PDGFB-based stem cell gene therapy increases bone strength in the mouse. Proc. Natl. Acad. Sci. USA.

[109] DiGiovanni C.W., Lin S.S., Baumhauer J.F., Daniels T., Younger A., Glazebrook M., Anderson J., Anderson R., Evangelista P., Lynch S.E. (2013). Recombinant human platelet-derived growth factor-BB and beta-tricalcium phosphate (rhPDGF-BB/β-TCP): An alternative to autogenous bone graft. J. Bone Jt. Surgery-Am. Vol..

[110] Daniels T.R., Younger A.S.E., Penner M.J., Wing K.J., Le I.L.D., Russell I.S., Lalonde K.-A., Evangelista P.T., Quiton J.D., Glazebrook M. (2015). Prospective randomized controlled trial of hindfoot and ankle fusions treated with rhPDGF-BB in combination with a β-TCP-collagen matrix. Foot Ankle Int..

[111] Coffin J.D., Homer-Bouthiette C., Hurley M.M. (2018). Fibroblast growth factor 2 and its receptors in bone biology and disease. J. Endocr. Soc..

[112] Charoenlarp P., Rajendran A.K., Iseki S. (2017). Role of fibroblast growth factors in bone regeneration. Inflamm. Regen..

[113] Kawaguchi H., Oka H., Jingushi S., Izumi T., Fukunaga M., Sato K., Matsushita T., Nakamura K. (2010). A local application of recombinant human fibroblast growth factor 2 for tibial shaft fractures: A randomized, placebo-controlled trial. J. Bone Miner. Res..

[114] Mayahara H., Ito T., Nagai H., Miyajima H., Tsukuda R., Taketomi S., Mizoguchi J., Kato K. (1993). In vivo stimulation of endosteal bone formation by basic fibroblast growth factor in rats. Growth Factors.

[115] Kawaguchi H., Kurokawa T., Hanada K., Hiyama Y., Tamura M., Ogata E., Matsumoto T. (1994). Stimulation of fracture repair by recombinant human basic fibroblast growth factor in normal and streptozotocin-diabetic rats. Endocrinology.

[116] Nakamura T., Hara Y., Tagawa M., Tamura M., Yuge T., Fukuda H., Nigi H. (1998). Recombinant human basic fibroblast growth factor accelerates fracture healing by enhancing callus remodeling in experimental dog tibial fracture. J. Bone Miner. Res..

[117] Kawaguchi H., Jingushi S., Izumi T., Fukunaga M., Matsushita T., Nakamura T., Mizuno K., Nakamura T., Nakamura K. (2007). Local application of recombinant human fibroblast growth factor-2 on bone repair: A dose–escalation prospective trial on patients with osteotomy. J. Orthop. Res..

[118] Xiao L., Liu P., Li X., Doetschman T., Coffin J.D., Drissi H., Hurley M.M. (2009). Exported 18-kDa isoform of fibroblast growth factor-2 is a critical determinant of bone mass in mice. J. Biol. Chem..

[119] Hurley M.M., Adams D.J., Wang L., Jiang X., Burt P.M., Du E., Xiao L. (2016). Accelerated fracture healing in transgenic mice overexpressing an anabolic isoform of fibroblast growth factor 2. J. Cell. Biochem..

[120] Naganawa T., Xiao L., Coffin J.D., Doetschman T., Sabbieti M.G., Agas D., Hurley M.M. (2008). Reduced expression and function of bone morphogenetic protein-2 in bones of Fgf2 null mice. J. Cell. Biochem..

[121] Van Gastel N., Stegen S., Stockmans I., Moermans K., Schrooten J., Graf D., Luyten F.P., Carmeliet G. (2014). Expansion of murine periosteal progenitor cells with fibroblast growth factor 2 reveals an intrinsic endochondral ossification program mediated by bone morphogenetic protein 2. Stem Cells.

[122] Hurley M.M., Tetradis S., Huang Y.-F., Hock J., Kream B.E., Raisz L.G., Sabbieti M.G. (1999). Parathyroid hormone regulates the expression of fibroblast growth factor-2 mRNA and fibroblast growth factor receptor mRNA in osteoblastic cells. J. Bone Miner. Res..

[123] Fei Y., Gronowicz G., M. Hurley M. (2013). Fibroblast Growth Factor-2, Bone Homeostasis and Fracture Repair. Curr. Pharm. Des..

[124] Fei Y., Xiao L., Doetschman T., Coffin D.J., Hurley M.M. (2011). Fibroblast Growth Factor 2 Stimulation of Osteoblast Differentiation and Bone Formation Is Mediated by Modulation of the Wnt Signaling Pathway. J. Biol. Chem..

[125] Wang X., Ito A., Sogo Y., Li X., Tsurushima H., Oyane A. (2009). Ascorbate–apatite composite and ascorbate–FGF-2–apatite composite layers formed on external fixation rods and their effects on cell activity in vitro. Acta Biomater..

[126] Rhee Y., Bivi N., Farrow E., Lezcano V., Plotkin L.I., White K.E., Bellido T. (2011). Parathyroid hormone receptor signaling in osteocytes increases the expression of fibroblast growth factor-23 in vitro and in vivo. Bone.

[127] Makrythanasis P., Temtamy S., Aglan M.S., Otaify G.A., Hamamy H., Antonarakis S.E. (2014). A novel homozygous mutation in FGFR3 causes tall stature, severe lateral tibial deviation, scoliosis, hearing impairment, camptodactyly, and arachnodactyly. Hum. Mutat..

[128] Ornitz D.M., Legeai-Mallet L. (2017). Achondroplasia: Development, pathogenesis, and therapy. Dev. Dyn..

[129] Rundle C.H., Miyakoshi N., Ramirez E., Wergedal J.E., Lau K.-H.W., Baylink D.J. (2002). Expression of the fibroblast growth factor receptor genes in fracture repair. Clin. Orthop. Relat. Res..

[130] Valverde-Franco G., Liu H., Davidson D., Chai S., Valderrama-Carvajal H., Goltzman D., Ornitz D.M., Henderson J.E. (2003). Defective bone mineralization and osteopenia in young adult FGFR3-/- mice. Hum. Mol. Genet..

[131] Su N., Sun Q., Li C., Lu X., Qi H., Chen S., Yang J., Du X., Zhao L., He Q. (2010). Gain-of-function mutation in FGFR3 in mice leads to decreased bone mass by affecting both osteoblastogenesis and osteoclastogenesis. Hum. Mol. Genet..

[132] Xie Y., Luo F., Xu W., Wang Z., Sun X., Xu M., Huang J., Zhang D., Tan Q., Chen B. (2017). FGFR3 deficient mice have accelerated fracture repair. Int. J. Biol. Sci..

[133] Su N., Li X., Tang Y., Yang J., Wen X., Guo J., Tang J., Du X., Chen L. (2016). Deletion of FGFR3 in osteoclast lineage cells results in increased bone mass in mice by inhibiting osteoclastic bone resorption. J. Bone Miner. Res..

[134] Yuan H., Yang Z., Li Y., Zhang X., De Bruijn J.D., De Groot K. (1998). Osteoinduction by calcium phosphate biomaterials. J. Mater. Sci. Mater. Med..

[135] Zwingenberger S., Nich C., Valladares R.D., Yao Z., Stiehler M., Goodman S.B. (2012). Recommendations and considerations for the use of biologics in orthopedic surgery. BioDrugs.

[136] Beuerlein M.J.S., McKee M.D. (2010). Calcium sulfates: What Is the evidence?. J. Orthop. Trauma.

[137] Rizzi S.C., Heath D.J., Coombes A.G.A., Bock N., Textor M., Downes S. (2001). Biodegradable polymer/hydroxyapatite composites: Surface analysis and initial attachment of human osteoblasts. J. Biomed. Mater. Res..

[138] Klein C.P.A.T., Driessen A.A., de Groot K., van den Hooff A. (1983). Biodegradation behavior of various calcium phosphate materials in bone tissue. J. Biomed. Mater. Res..

[139] Daculsi G., Legeros R.Z., Nery E., Lynch K., Kerebel B. (1989). Transformation of biphasic calcium phosphate ceramics in vivo: Ultrastructural and physicochemical characterization. J. Biomed. Mater. Res..

[140] Chow L.C., Takagi S. (2001). A Natural Bone Cement - A Laboratory Novelty Led to the Development of Revolutionary New Biomaterials. J. Res. Natl. Inst. Stand. Technol..

[141] Xu H.H.K., Weir M.D., Burguera E.F., Fraser A.M. (2006). Injectable and macroporous calcium phosphate cement scaffold. Biomaterials.

[142] Weir M.D., Xu H.H.K. (2010). Osteoblastic induction on calcium phosphate cement-chitosan constructs for bone tissue engineering. J. Biomed. Mater. Res. Part A.

[143] Kim S.-S., Park M.S., Gwak S.-J., Choi C.Y., Kim B.-S. (2006). Accelerated bonelike apatite growth on porous polymer/ceramic composite scaffolds in vitro. Tissue Eng..

[144] Jeon O.H., Panicker L.M., Lu Q., Chae J.J., Feldman R.A., Elisseeff J.H. (2016). Human iPSC-derived osteoblasts and osteoclasts together promote bone regeneration in 3D biomaterials. Sci. Rep..

[145] Kim S.-S., Park M.S., Jeon O., Choi C.Y., Kim B.-S. (2006). Poly(lactide-co-glycolide)/hydroxyapatite composite scaffolds for bone tissue engineering. Biomaterials.

[146] Wang P., Song Y., Weir M.D., Sun J., Zhao L., Simon C.G., Xu H.H.K. (2016). A self-setting iPSMSC-alginate-calcium phosphate paste for bone tissue engineering. Dent. Mater..

[147] Hench L.L., Splinter R.J., Allen W.C., Greenlee T.K. (1971). Bonding mechanisms at the interface of ceramic prosthetic materials. J. Biomed. Mater. Res..

[148] Moimas L., Biasotto M., Di Lenarda R., Olivo A., Schmid C. (2006). Rabbit pilot study on the resorbability of three-dimensional bioactive glass fibre scaffolds. Acta Biomater..

[149] Allan I., Newman H., Wilson M. (2001). Antibacterial activity of particulate Bioglass^®^ against supra- and subgingival bacteria. Biomaterials.

[150] Stoor P., Söderling E., Salonen J.I. (1998). Antibacterial effects of a bioactive glass paste on oral microorganisms. Acta Odontol. Scand..

[151] Coraça-Huber D.C., Fille M., Hausdorfer J., Putzer D., Nogler M. (2014). Efficacy of antibacterial bioactive glass S53P4 against S. aureus biofilms grown on titanium discs in vitro. J. Orthop. Res..

[152] Drago L., Romanò D., De Vecchi E., Vassena C., Logoluso N., Mattina R., Romanò C.L. (2013). Bioactive glass BAG-S53P4 for the adjunctive treatment of chronic osteomyelitis of the long bones: An in vitro and prospective clinical study. BMC Infect. Dis..

[153] Aurégan J.-C. (2015). Bioactive glass for long bone infection: A systematic review. Injury.

[154] Baino F., Hamzehlou S., Kargozar S. (2018). Bioactive glasses: Where are we and where are we going?. J. Funct. Biomater..

[155] George A., Veis A. (2008). Phosphorylated proteins and control over apatite nucleation, crystal growth, and inhibition. Chem. Rev..

[156] Landis W.J., Silver F.H. (2009). Mineral deposition in the extracellular matrices of vertebrate tissues: Identification of possible apatite nucleation sites on type I collagen. Cells. Tissues. Organs.

[157] Nudelman F., Pieterse K., George A., Bomans P.H.H., Friedrich H., Brylka L.J., Hilbers P.A.J., de With G., Sommerdijk N.A.J.M. (2010). The role of collagen in bone apatite formation in the presence of hydroxyapatite nucleation inhibitors. Nat. Mater..

[158] Itoh S., Kikuchi M., Koyama Y., Takakuda K., Shinomiya K., Tanaka J. (2004). Development of a hydroxyapatite/collagen nanocomposite as a medical device. Cell Transplant..

[159] Walsh W.R., Oliver R.A., Christou C., Lovric V., Walsh E.R., Prado G.R., Haider T. (2017). Critical size bone defect healing using collagen-calcium phosphate bone graft materials. PLoS ONE.

[160] Li C., Cheung T.F., Fan V.C., Sin K.M., Wong C.W.Y., Leung G.K.K. (2017). Applications of three-dimensional printing in surgery. Surg. Innov..

[161] Bauermeister A.J., Zuriarrain A., Newman M.I. (2016). Three-dimensional printing in plastic and reconstructive surgery. Ann. Plast. Surg..

[162] Fang C., Cai H., Kuong E., Chui E., Siu Y.C., Ji T., Drstvenšek I. (2019). Surgical applications of three-dimensional printing in the pelvis and acetabulum: From models and tools to implants. Unfallchirurg.

[163] Guvendiren M., Molde J., Soares R.M.D., Kohn J. (2016). Designing biomaterials for 3D printing. ACS Biomater. Sci. Eng..

[164] Trombetta R., Inzana J.A., Schwarz E.M., Kates S.L., Awad H.A. (2017). 3D printing of calcium phosphate ceramics for bone tissue engineering and drug delivery. Ann. Biomed. Eng..

[165] Anitha A., Menon D., Sivanarayanan T.B., Koyakutty M., Mohan C.C., Nair S.V., Nair M.B. (2017). Bioinspired composite matrix containing hydroxyapatite–silica core–shell nanorods for bone tissue engineering. ACS Appl. Mater. Interfaces.

[166] Turner R.J., Renshaw J.C., Hamilton A. (2017). Biogenic hydroxyapatite: A new material for the preservation and restoration of the built environment. ACS Appl. Mater. Interfaces.

[167] Richards D.J., Coyle R.C., Tan Y., Jia J., Wong K., Toomer K., Menick D.R., Mei Y. (2017). Inspiration from heart development: Biomimetic development of functional human cardiac organoids. Biomaterials.

[168] Löffler S., Seyock S., Nybom R., Jacobson G.B., Richter-Dahlfors A. (2016). Electrochemically triggered release of acetylcholine from scCO2 impregnated conductive polymer films evokes intracellular Ca2+ signaling in neurotypic SH-SY5Y cells. J. Control. Release.

[169] Wang T., Wang D., Liu J., Feng B., Zhou F., Zhang H., Zhou L., Yin Q., Zhang Z., Cao Z. (2017). Acidity-triggered ligand-presenting nanoparticles to overcome sequential drug delivery barriers to tumors. Nano Lett..

[170] Hang C., Zou Y., Zhong Y., Zhong Z., Meng F. (2017). NIR and UV-responsive degradable hyaluronic acid nanogels for CD44-targeted and remotely triggered intracellular doxorubicin delivery. Colloids Surf. B Biointerfaces.

[171] He M., Wang Q., Shi Z., Xie Y., Zhao W., Zhao C. (2017). Inflammation-responsive self-regulated drug release from ultrathin hydrogel coating. Colloids Surf. B Biointerfaces.

[172] Minkowitz R.B. (2007). Removal of painful orthopaedic implants after fracture union. J. Bone Jt. Surg..

[173] Zhao D., Witte F., Lu F., Wang J., Li J., Qin L. (2017). Current status on clinical applications of magnesium-based orthopaedic implants: A review from clinical translational perspective. Biomaterials.

